# Copulation or sensory cues from the female augment Fos expression in arginine vasopressin neurons of the posterodorsal medial amygdala of male rats

**DOI:** 10.1186/1742-9994-11-42

**Published:** 2014-06-02

**Authors:** Shantala Arundathi Hari Dass, Ajai Vyas

**Affiliations:** 1 School of Biological Sciences, Nanyang Technological University, 60 Nanyang Drive, Nanyang 637551, Republic of Singapore

**Keywords:** Affiliation, Mating, Neuropeptide, Nonapeptide, Pheromone, Sexual behavior, Social behavior, Testosterone, Vasotocin

## Abstract

**Background:**

The posterodorsal part of the medial amygdala is essential for processing reproductively salient sensory information in rodents. This is the initial brain structure where information from olfactory system and male hormones intersect. The neurochemical identity of the neurons participating in the sensory processing in medial amygdala remains presently undetermined. Many neurons in this brain structure express arginine vasopressin in a testosterone-dependent manner, suggesting that this neuropeptide is maintained by the androgenic milieu.

**Method:**

Here we use Fos, a protein expressed by recently active neurons, to quantify activation of arginine vasopressin neurons after exposure to odor from physically inaccessible female. We compare it to mating with accessible female and to reproductively innocuous odor.

**Results:**

We show that inaccessible female activate arginine vasopressin neurons in the male posterodorsal medial amygdala. The magnitude of activation is not further enhanced when physical access with resultant mating is granted, even though it remains undetermined if same population of AVP neurons is activated by both inaccessible female and copulation. We also show that arginine vasopressin activation cannot be fully accounted for by mere increase in the number of Fos and AVP neurons.

**Conclusion:**

These observations posit a role for the medial amygdala arginine vasopressin in reproductive behaviors, suggesting that these neurons serve as integrative node between the hormonal status of the animal and the availability of reproductive opportunities.

## Introduction

The medial amygdala (MeA) plays an important role during male reproductive behavior in the rodents [1]. Lesions of the MeA reduce reproductive behavior in hamsters [2], rats [3] and gerbils [4]. In hamsters, the anterior MeA is involved in discrimination of conspecific odor from same-sex versus opposite-sex donors, while the posterodorsal MeA is selectively activated by opposite-sex conspecifics [5]. Fiber-sparing lesions of anterior MeA in this species reduce number of Fos immunoreactive cells in the posterodorsal MeA and efferent forebrain regions [6], suggesting a unidirectional flow of chemosensory information through anterior MeA to its downstream targets. In male rats, MeA lesions drastically reduce penile erections in response to an inaccessible estrous female [7]. Interestingly, such lesions do not affect reflexive erections in response to penile sheath retraction. These observations suggest that MeA involvement in male reproductive behavior is restricted to motivation and not to the downstream initiation of mating in this species.

MeA is a sexually dimorphic structure (reviewed in [8]), characterized by more neurons and larger neuronal soma in males compared to females [9,10]. Important from the perspective of this report, male MeA also contains sunstantial number of extra-hypothalamic parvocellular population of arginine vasopressin (AVP) neurons [11]. Testosterone is required for sexual dimorphism of the MeA neurons [8] and also for AVP expression in the MeA [12,13]. The essential nature of testosterone is further supported by the observations that antagonism of androgen receptors in the MeA inhibits penile erection in male rats in presence of estrus females [14]; an effect reversed by testosterone implants within the MeA of castrates [15,16].

Further evidence suggests that the contribution of the MeA to reproductive behavior is anchored in its ability to integrate pheromonal information with hormonal milieu. Soiled bedding from females increases MeA-Fos in male mice with or without aromatase [17] and in testosterone-primed gonadectomized rats [18,19]. Since Fos is regarded as a proxy for recent neuronal activity [20], this observation suggests that pheromones enhance the activity of MeA neurons. Similarly, female vaginal fluid increases Fos expression in the MeA of mandarin voles [21]. These observations provide correlational support for activation of the MeA in pheromonal processing. Experiments in Syrian hamster further strengthen this. Mating in this species requires intact ability to smell female pheromones and presence of testicular testosterone; absence of either countermands copulation. Interestingly, implantation of testosterone in the MeA of castrates can reinitiate copulatory behavior [22]. Yet, the ability of the testosterone to rescue effects of the castration is dependent on olfactory inputs, such that surgical removal of olfactory bulbs renders testosterone implants ineffective [22,23]. Since both olfactory inputs afferent to MeA and testosterone within MeA are required for the rescue, it suggests that MeA integrates information from sensory environment and internal androgenic milieu.

Despite the role of the MeA in processing of reproductively salient sensory cues, the neurochemical identity of the pertinent cell groups is yet undetermined. As mentioned before, many MeA neurons also express AVP in a testosterone dependent manner. This is important because the AVP mediates several social and sexual behaviors, e.g. monogamy in voles [24] and social recognition of juveniles in male rats [25]. Moreover, AVP neurons are activated during copulation in bed nucleus of stria terminalis [26], a brain region with significant neuro-architectural similarity to the MeA.

In view of androgen-dependent expression of the MeA-AVP and the role of the testosterone in the pheromonal processing, we hypothesized that sensory cues from females selectively activate AVP producing neurons in the MeA. We tested this hypothesis by quantifying co-labeling of AVP and Fos, an immediate early gene product that marks recently activated neurons, post-exposure to rabbit odor or inaccessible estrus female or copulation. Posteroventral and posterodorsal sub-nuclei of MeA were quantified separately in view of their disparate neurochemistry and differential involvement in processing of olfactory signals [27].

## Results and discussion

**Figure 1:**
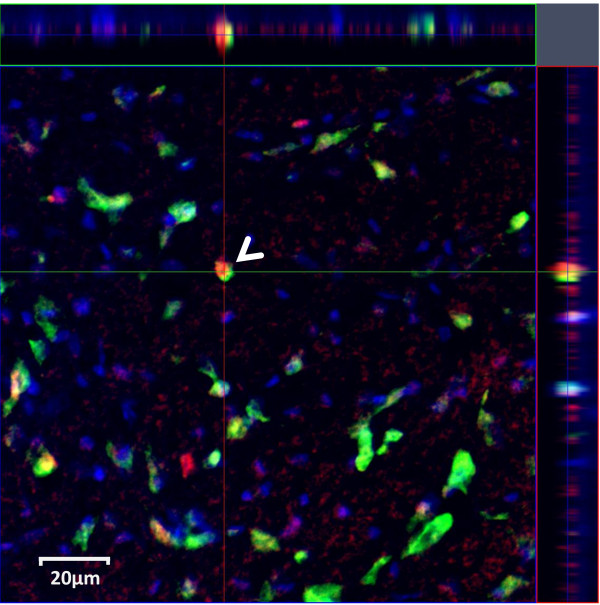
**Representative image depicting colabeling of Fos and AVP antigens in MePD.** Fos is stained in red color (DyLight549, emission = 568 nm) and AVP is in green (Fluorescein, emission = 517 nm). DAPI is in blue. Inset on top and right portions depict confocal slice along planes marked by red and green lines, respectively. White arrow highlights a colabelled neuron expressing AVP and FOS.

Figure 1 depicts a representative image acquired after histological staining for arginine vasopressin immunoreactive (AVP-ir) and Fos immunoreactive (Fos-ir) neurons in the MePD. Total number of DAPI cells imaged did not significantly differ between the brain regions or the experimental treatments (ANOVA: *p* > 0.29).

### Reproductive stimuli suppressed number of Fos-ir neurons in the MePV, but increased it in the MePD

We utilized repeated measure ANOVA to compare number of Fos-ir neurons in MePV and MePD across experimental treatments. Main effect of the experimental treatments reached statistical significance (F_(2,14)_ = 4.44; *p* =0.032). ANOVA revealed significant differences for the main effect of the sub-nuclei (MePD > MePV, 59.2% change in marginal means; F_(1,14)_ = 35.46; *p* < 0.001) and the interaction (F_(2,14)_ = 29.21; *p* < 0.001).

**Figure 2:**
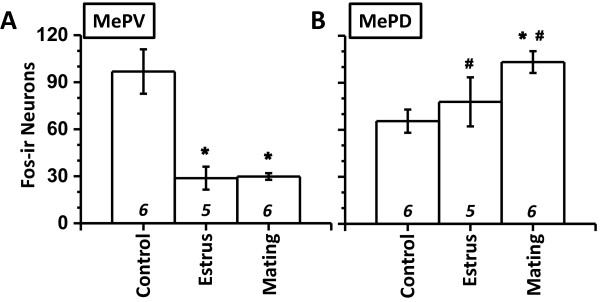
**Fos expression in MePV and MePD.** Ordinate depicts call counts of Fos-ir neurons () in MePV **(A)** and MePD **(B)**. N is indicated above abscissa (*italics*). *, *p* < 0.05, post-hoc comparison between experimental treatments within a particular sub-nuclei. #, *p* < 0.05, post-hoc paired comparison between sub-nuclei for a particular experimental treatment. Mean ± SEM.

Specifically in the MePV, animals exposed to an estrus female or copulation contained a significantly reduced number of Fos-ir neurons compared to a reproductively neutral stimulus (Figure 2A, > 65% reduction; post-hoc LSD test: *p* ≤ 0.001). It is not obvious whether this represents a reduction in Fos-ir due to reproductive salient stimuli or if rabbit urine increased Fos-ir above the unquantified quiescent baseline. No statistical difference was evident between the males exposed to estrus females with or without the opportunity of mating (Figure 2A; *p* = 0.181).

In contrast to the MePV, the number of Fos-ir neurons in the MePD exhibited significant increase after copulation, compared to rabbit odor (Figure 2B, 57% increase; *p* < 0.05). Exposure to an estrous female did not result in a significant increase (*p* = 0.259). The number of Fos-ir neurons was greater in the MePD compared to the MePV when animals were treated with reproductive stimuli (post-hoc paired t-test: *p* < 0.05) and not statistically different during control stimulus (|t_5_| = 2.35, *p* = 0.066).

### MePD contained greater number of AVP-ir neurons

**Figure 3:**
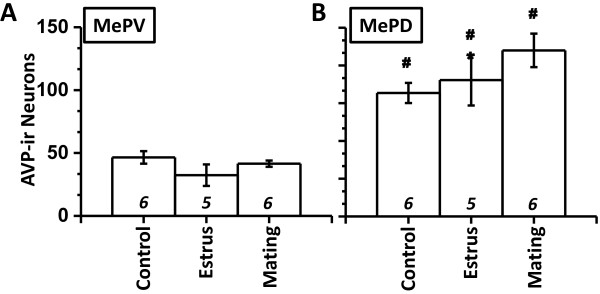
**AVP expression in MePV and MePD.** Cell counts of AVP-ir neurons () in MePV **(A)** and MePD **(B)**. N is indicated above abscissa (*italics*). *, *p* < 0.05, post-hoc comparison between experimental treatments within a particular sub-nuclei. #, *p* < 0.05, post-hoc paired comparison between sub-nuclei for a particular experimental treatment.

ANOVA revealed a significant main effect of the brain regions (F_(1,14)_ = 177.3; *p* < 0.001). Main effect of the experimental treatment did not reach statistical significance (F_(2,14)_ = 2.13; *p* < 0.156). Interaction between treatment and sub-nuclei revealed significant differences (F_(2,14)_ = 4.58; *p* < 0.030). Marginal mean of AVP-ir number for MePD was substantially greater than that for MePV (181% more, MePD relative to MePV; *p* < 0.001). Similarly for all experimental treatments, AVP-ir in MePD surpassed observations in MePV (Figure 3; post-hoc paired Student’s t-test: *p* ≤ 0.001). In view of significant interaction, we further investigated AVP-ir values between experimental groups in MePV and MePD (post-hoc LSD test). Significant inter-group differences were not observed in MePV (Figure 3A; *p* = 0.16). In MePD, no significant differences were observed between control and estrous group (Figure 3B; *p* = 0.482). In contrast, males exposed to estrus females with opportunity to mate exhibited significantly greater number of AVP-ir compared to control stimuli (27% increase; *p* < 0.05).

### Reproductive stimuli increased number of colabeled neurons in MePD, but not in MePV

ANOVA revealed significant main effects of experimental treatments (F_(2,14)_ = 27.28; *p* < 0.001) and of sub-nuclei (F_(1,14)_ = 373.72; *p* < 0.0001). Interaction between treatments and sub-nuclei was also highly significant (F_(2,14)_ = 73.10; *p* < 0.001).

**Figure 4:**
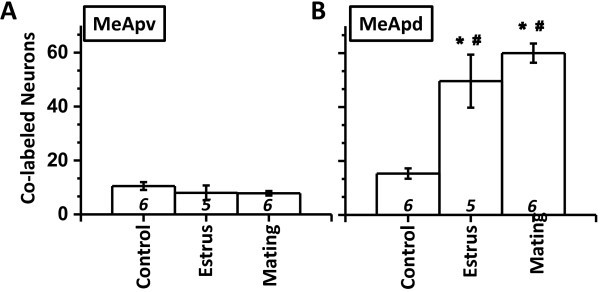
**Colabeling in MePV and MePD.** Cell counts of colabeled neurons () in MePV **(A)** and MePD **(B)**. *, *p* < 0.05, post-hoc comparison between experimental treatments within a particular sub-nuclei. #, *p* < 0.05, post-hoc paired comparison between sub-nuclei for a particular experimental treatment.

Consistent with lesser number of AVP-ir neurons in the MePV, this sub-nuclei also contained lower number of colabeled neurons (AVP-ir and Fos-ir; marginal mean: MePV = 8.33 ± 1.04, MePD = 38.88 ± 5.56). Across all experimental groups, the MePD contained greater number of colabeled neurons than MePV (Figure 4; post-hoc paired Student’s t-test: *p* < 0.01). Within MePV, experimental treatments did not significantly change the number of colabeled neurons (Figure 4A; *p* > 0.3).

In the MePD, exposure to estrus females robustly increased number of colabeled neurons compared to the control stimuli (Figure 4B, 233% increase; post-hoc LSD test: *p* < 0.00001). Similarly, mating with females also increased number of colabeled neurons in the male MePD (300% increase; *p* < 0.00001).

### Reproductively salient stimuli specifically activated AVP-ir neurons in the MePD

In the MePD, >45% of Fos-ir neurons activated by either estrus female or mating expressed AVP (Table 1; ≈ 3-fold increase compared to rabbit odor). Similarly, >58% of all imaged MePD AVP-ir neurons also expressed Fos (Table 1) after exposure to female or copulation. Data described above demonstrates an increase in MePD colabeled neurons. To analyze if the number of colabeled neurons were reflective of more Fos-ir and AVP-ir, we compared observed and expected (based on mathematical product of AVP-ir and Fos-ir frequencies) values using a repeated measure ANOVA.

**Table 1:** Mean ± SeM values pertaining to co-labeled AVP-ir and Fos-ir cells

		**Mean ± SeM**		
		**Rabbit (n = 6)**	**Estrous (n = 5)**	**Copulation (n = 6)**
*Percentage of Fos-ir cells that were also AVP-ir*				
MePV		23.6 ± 3.9	23.4 ± 6.3	19.2 ± 2.3
MePD		5.5 ± 1.0	45.6 ± 2.8*#	47.0 ± 3.6*#
*Percentage of AVP-ir cells that were also Fos-ir*				
MePV		11.8 ± 1.8	28.2 ± 5.7*	27.2 ± 4.1*
MePD		24.3 ± 3.9 #	64.5 ± 4.3*#	58.4 ± 1.6*#

Main effect of intra-animal brain regions (MePV versus MePD): F_(1,14)_ > 8.2; *p* < 0.001.

Main effect of inter-animals treatments: F_(2,14)_ > 3.5; *p* < 0.001.

Interaction: F_(2,14)_ > 23.7; *p* < 0.001.

*p < 0.05, post-hoc comparison between experimental treatments within a particular sub-nuclei. #, *p* < 0.001, post-hoc paired comparison between sub-nuclei for a particular experimental treatment.

**Figure 5:**
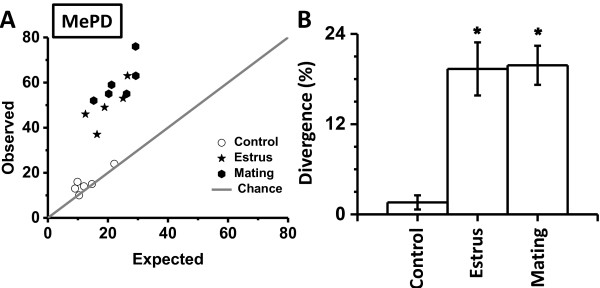
**Departure of observed colabeling from theoretical prediction.** Expected and observed values of colabeled neurons in the MePD **(A)**. Expected probability (abscissa) was calculated as product of individual probabilities for FOS and AVP neurons. The diagonal gray line from the origin depicts chance level (expected probability equals observed probability). Divergence of observed values from the chance **(B)**. Divergence was calculated for each Cartesian point in panel A by dividing (x-y)^2^ with (x + y)^2^. Divergence is expressed as percentage on the ordinate. *p < 0.05, post-hoc comparison between experimental treatments.

In case of the MePV, ANOVA revealed that the interaction between treatment and the observed/expected values were not significantly different (F_(2,14)_ = 1.94; *p* < 0.181). On the other hand, observed values for the MePD were substantially divergent from the expectations (main effect: F_1,14)_ = 348.93, *p* < 0.0001; 119% difference, expected < observed), suggesting a selective Fos activation of AVP-ir neurons. Amongst experimental groups, animals exposed to reproductively salient stimuli exhibited greater departure of the observed values from the expectations (Figure 5A; chance is depicted by diagonal gray line). We further recapitulated this departure by calculating a divergence scale that was indifferent to the distance of the expected/observed Cartesian points from the origin. For each point in Figure 5A, we calculated divergence by dividing (x-y)^2^ with (x + y)^2^, expressed in percentage (Figure 5B). A one-way ANOVA revealed that exposure to reproductively salient stimuli significantly enhanced divergence between expected and observed values (F_(2,14)_ = 18.45, *p* < 0.001; Figure 5B; > 350% increase; *p* < 0.001).

Amongst the various nuclei of the extended amygdala, the MeA is especially important for the appetitive aspects of the reproduction because it is the initial site during pheromonal processing where main and accessory olfactory information intersects [5,28,29]. It robustly expresses androgen receptors and is sexually dimorphic in the structure [30-32]. The androgen responsiveness suggests that the MEA has access to and can plausibly integrate both the internal hormonal milieu and external pheromonal environment. Lesions of this structure ablate penile erections in male rats in response to an inaccessible female [23], while leaving reflexive erections intact [7]. Importance of MeA efferents for mating can be ascertained by the observation that simultaneous unilateral MeA lesion coupled with contralateral medial preoptic lesion ablates mating behavior [33]. The MeA also shares bidirectional innervation with bed nucleus of stria terminalis (BNST). For example, in male hamsters after exposure to a female odor, greater number of MePD neurons colabeled with Fos and a retrograde tracer injected in the BNST [34]. This demonstrates that female odors increases activity in BNST-projecting neurons of the MePD, compared to an odor from male conspecifics. The greater communication between MePD and the BNST is consistent with the ontological and hodological similarities between these brain regions [35].

Despite the importance of neurons in the MeA in the processing of reproductively salient cues, neurochemical identity of the activated neurons is still undetermined. Data presented in this report shows that the exposure to a physically inaccessible female rat can selectively activate AVP neurons in the MePD, in contrast to the MePV or in contrast to reproductively innocuous rabbit urine. Moreover, the extent of AVP neurons being activated cannot be explained by arithmetic changes in AVP or Fos neurons, thus suggesting that AVP neurons are activated in a non-random manner. While the present report examined two sub-nuclei of the MeA, it is possible that other MeA sub-nuclei or other parts of the social brain network are also activated during exposure to the reproductively salient stimuli.

We demonstrate a rapid increase in the density of Fos neurons in the MePD after exposure to an inaccessible female or post-copulation. This suggests that Fos in the MePD is dynamically regulated by the presence of sensory cues. Yet, this increase in Fos density is not sufficient to explain the full extent of MePD-AVP activation. This is substantiated by the fact that a greater number of active neurons express AVP and a greater fraction of the AVP neurons become active. Thus, AVP neurons are activated during exposure to sexually salient environmental signals. This is consistent with the prior observations in desert finches expressing vasotocin, a neuropeptide homologous to the AVP. This bird is an opportunistic breeder similar to the rat. In this species co-housing with females enhances activation of the pre-existing vasotocin neurons in the bed nucleus of stria terminalis of the males, compared to the unisexual group of males [36].

The extent of MePD-AVP activation does not seem to be affected by presence or absence of the physical contact or mating itself. In other words, sensory signals of a physically inaccessible female and the actual mating induce equivalent amount of AVP activation. It should be however noted that experiments described in this report cannot differentiate if same or different population of MePD-AVP neurons are activated by inaccessible female and copulation. Localized manipulations in MeA does increase reproductive motivation of the males even when females are inaccessible or even when soiled bedding or vaginal fluids are used instead of the females [30]. Testosterone implants in the MeA rescue the effects of castration on appetitive aspects of the reproductive behavior. It is not clear if testosterone in these cases directly activates androgen receptor and/or is aromatized to estradiol and subsequently binds to estrogen receptors. Mice lacking aromatase gene exhibit MeA-Fos activation comparable to the wild-type individuals when exposed to female odors [17], suggesting direct role for the androgen receptors. These observations and data in present report suggest, but do not prove, that MePD is involved in processing of information with no additional role in mating. It should be noted that, contrary to the suggestion of non-additional role of the MeA testosterone in consummatory reproductive behavior, androgen receptor occupancy in MeA does facilitate intromission and ejaculation if simultaneous estrogen receptor binding is available throughout the brain and the periphery [37].

AVP neurons mediate social and sexual behaviors in a variety of species and paradigms. Relevant examples include social recognition in rats [38-40] and development of pair bonding in monogamous voles [24]. Homologous neuropeptides are involved in flocking and territoriality in birds [41]; and in mate recognition and mating in nematode *Caenorhabditis elegans*[42]. AVP neurons in medial bed nucleus of stria terminalis are also activated during copulation in mice [26,43]. While these strands of evidences suggest a pervasive role for the AVP in a variety of social and sexual behaviors, it is currently unknown if the sexual sensory cues themselves activate AVP neurons. In this backdrop, we show that exposure to inaccessible females is able to activate AVP neurons in MePD, the actual act of copulation not being obligatory. This is consistent with the prior observations that testosterone acting within the MeA promotes sexual arousal to the odor of female rats, without any apparent effect on mating behavior itself.

Reproductive investment and testosterone levels in animals are frequently calibrated to incipient metabolic conditions and mating opportunities [44,45]. It is plausible that AVP within the MePD serves as integrative node between reproductive status of the animal (signaled by testosterone mediated expression of AVP) and availability of reproductive opportunities (signaled by activation of AVP neurons by sexual pheromones). Related to this, social isolation after weaning reduces the volume of MeA and blunts the sexual behavior in rats [46]. Similarly, ageing reduces MeA-AVP and AVP innervation to brain regions efferent to the MeA [47].

## Conclusion

In conclusion, data presented here show that AVP neurons in MePD are activated during the processing of reproductive cues. It is likely that these neurons play critical role in the mediation of pheromone-directed reproductive behaviors. In addition this report provides support to the role of extra-hypothalamic AVP neurons in reproductive and affiliative behaviors.

## Methods

### Animals

Wistar rats (47–50 days old) were obtained from the vivarium at National University of Singapore. Animals (housed 2/cage) were maintained on 12 hour light–dark cycle with *ad libitum* food and water (lights on at 0700 hours). The Nanyang Technological University institutional animal care and use committee reviewed and approved all procedures. These procedures are compliant with the NIH guidelines. All the male and female subjects used in this paper were sexually naïve at the start of the experiment.

### Exposure to reproductive stimuli

Males were habituated for ten successive days ((ten minutes each day, between 1100 and 1400 hours) in a rectangular arena, in which they would eventually receive the stimuli (46 × 9 cm; 15 cm high). On the day of exposure males were shifted into the procedure room just before the beginning of the light phase (7 AM). After a four hour rest period they were exposed to either a physically inaccessible estrus female behind translucent perforated plastic partition (N = 5) or allowed to mate with an accessible receptive female (N = 6). Females were allowed to explore the entire arena for two hours before the start of the trial, during which time they placed urine marks in the arena. To control for novelty of the odor, a third group of males was exposed to rabbit urine on an inaccessible towel (N = 6). All animals were sacrificed two hour after the onset of stimulus exposure.

Naturally cycling females were used as the stimulus. Estrus phase was determined using examination of vaginal lavage, obtained by gentle flushing of cells from vaginal lining using 20 μl buffered saline (between 1030 and 1100 hours). Unstained lavages were examined on a glass slide using 20× magnification. Females in estrus were identified by presence of cornified cells and absence of nucleated cells.

### Histological staining

Animals were deeply anaesthetized and transcardially perfused with 4% paraformaldehyde. Free floating brain sections (40 μm thick) were incubated in a cocktail of primary antibodies for 72 hours at 4°C (guinea pig anti-AVP, 1:500, Bachem; and, rabbit anti-Fos, 1:100, Santa Cruz Biotenchology). This was followed by incubation with secondary antibodies at room temperature for 2 hours (biotinylated anti guinea pig; 1: 200 + anti rabbit-DyLight 549;1:200; obtained from Vector Laboratories). The biotinylated antibody signal was developed using Vectastain elite ABC kit (Vector Laboratories) and tyramide signal amplification system (Perkin Elmer). Sections were counter stained with DAPI for 1 minute.

Brain sections between Bregma levels −2.76 mm and −3.24 mm (Interaural 7.28 to 7.08) were selected for analysis. Sections were imaged at 40× magnification and 1.2× digital zoom using a confocal microscope (optically sliced at 4 μm, three set of stacks per animal, Carl Zeiss LSM 710). Neurons positive for DAPI, Fos and AVP were counted. Scores were cumulated per animal.

### Calculation of observed and expected frequencies

We calculated the expected probability of encountering colabeled neurons by multiplying individual probabilities of AVP-ir and Fos-ir neurons. Individual probabilities for AVP-ir were calculated by division of number of AVP-ir neurons with total number of DAPI positive neurons counted (i.e. probability that a particular DAPI positive neuron will be also be AVP-ir). Individual probabilities for Fos-ir were also counted in the similar manner. A product of these probabilities defines the baseline expectation of colabeling by mere chance and assuming biological independence between Fos and AVP activation. The observed numbers of the colabeled cells were compared to the expected baseline, with null hypothesis of colabeling being a mere mathematical coincidence (adapted from [48]).

### Statistics

Repeated measures analysis of variance (ANOVA) was used to quantify statistical significance for main effects and interactions. In case of within-subject comparisons, paired Student’s t-test was employed for post-hoc significance testing. In case of between-subject comparisons, LSD test was used. Values reported are mean ± SEM.

## Competing interests

The authors declare that they have no competing interests.

## Authors’ contribution

SAHD performed all the experiments. AV and SAHD designed the experiments. AV and SAHD analysed the data. AV wrote the paper. Both authors read and approved the final manuscript.

## References

[B1] Greco B Edwards DA Michael RP Clancy AN Androgen receptors and estrogen receptors are colocalized in male rat hypothalamic and limbic neurons that express Fos immunoreactivity induced by mating Neuroendocrinology 1998 67 18 28 10.1159/000054294 9485165

[B2] Lehman MN Winans SS Powers JB Medial nucleus of the amygdala mediates chemosensory control of male hamster sexual behavior Science 1980 210 557 60 10.1126/science.7423209 7423209

[B3] Kondo Y Lesions of the medial amygdala produce severe impairment of copulatory behavior in sexually inexperienced male rats Physiol Behav 1992 51 939 43 10.1016/0031-9384(92)90074-C 1615054

[B4] Heeb MM Yahr P Cell-body lesions of the posterodorsal preoptic nucleus or posterodorsal medial amygdala, but not the parvicellular subparafascicular thalamus, disrupt mating in male gerbils Physiol Behav 2000 68 317 31 10.1016/S0031-9384(99)00182-1 10716541

[B5] Maras PM Petrulis A Chemosensory and steroid-responsive regions of the medial amygdala regulate distinct aspects of opposite-sex odor preference in male Syrian hamsters Eur J Neurosci 2006 24 3541 52 10.1111/j.1460-9568.2006.05216.x 17229102

[B6] Maras PM Petrulis A The anterior medial amygdala transmits sexual odor information to the posterior medial amygdala and related forebrain nuclei Eur J Neurosci 2010 32 469 82 10.1111/j.1460-9568.2010.07289.x 20704594

[B7] Kondo Y Sachs BD Sakuma Y Importance of the medial amygdala in rat penile erection evoked by remote stimuli from estrous females Behav Brain Res 1998 91 215 22 9578453

[B8] Cooke BM Steroid-dependent plasticity in the medial amygdala Neuroscience 2006 138 997 1005 10.1016/j.neuroscience.2005.06.018 16330154

[B9] Mizukami S Nishizuki M Arai Y Sexual difference in nuclear volume and its ontogeny in the rat amygdala Exp Neurol 1983 79 569 75 10.1016/0014-4886(83)90235-2 6822281

[B10] Cooke BM Tabibnia G Breedlove SM A brain sexual dimorphism controlled by adult circulating androgens Proc Natl Acad Sci U S A 1999 96 7538 40 10.1073/pnas.96.13.7538 10377450 PMC22121

[B11] Wang Z De Vries GJ Androgen and estrogen effects on vasopressin messenger RNA expression in the medial amygdaloid nucleus in male and female rats J Neuroendocrinol 1995 7 827 31 10.1111/j.1365-2826.1995.tb00722.x 8748118

[B12] DeVries GJ Buijs RM Van Leeuwen FW Caffe AR Swaab DF The vasopressinergic innervation of the brain in normal and castrated rats J Comp Neurol 1985 233 236 54 10.1002/cne.902330206 3882778

[B13] Auger CJ Coss D Auger AP Forbes-Lorman RM Epigenetic control of vasopressin expression is maintained by steroid hormones in the adult male rat brain Proc Natl Acad Sci 2011 108 4242 7 10.1073/pnas.1100314108 21368111 PMC3053981

[B14] Bialy M Nikolaev-Diak A Kalata U Nikolaev E Blockade of androgen receptor in the medial amygdala inhibits noncontact erections in male rats Physiol Behav 2011 103 295 301 10.1016/j.physbeh.2011.02.003 21315100

[B15] Bialy M Sachs BD Androgen implants in medial amygdala briefly maintain noncontact erection in castrated male rats Horm Behav 2002 42 345 55 10.1006/hbeh.2002.1821 12460594

[B16] Cooke BM Breedlove SM Jordan CL Both estrogen receptors and androgen receptors contribute to testosterone-induced changes in the morphology of the medial amygdala and sexual arousal in male rats Horm Behav 2003 43 336 46 10.1016/S0018-506X(02)00047-8 12694644

[B17] Aste N Honda S Harada N Forebrain Fos responses to reproductively related chemosensory cues in aromatase knockout mice Brain Res Bull 2003 60 191 200 10.1016/S0361-9230(03)00035-2 12754080

[B18] Bressler SC Baum MJ Sex comparison of neuronal Fos immunoreactivity in the rat vomeronasal projection circuit after chemosensory stimulation Neuroscience 1996 71 1063 72 10.1016/0306-4522(95)00493-9 8684610

[B19] Paredes RG Lopez ME Baum MJ Testosterone augments neuronal Fos responses to estrous odors throughout the vomeronasal projection pathway of gonadectomized male and female rats Horm Behav 1998 33 48 57 10.1006/hbeh.1998.1435 9571013

[B20] Hoffman GE Smith MS Verbalis JG c-Fos and related immediate early gene products as markers of activity in neuroendocrine systems Front Neuroendocrinol 1993 14 173 213 10.1006/frne.1993.1006 8349003

[B21] He F Wu R Yu P Study of Fos, androgen receptor and testosterone expression in the sub-regions of medial amygdala, bed nucleus of stria terminalis and medial preoptic area in male mandarin voles in response to chemosensory stimulation Behav Brain Res 2014 258 65 74 24129216 10.1016/j.bbr.2013.10.004

[B22] Wood RI Coolen LM Integration of chemosensory and hormonal cues is essential for sexual behaviour in the male Syrian hamster: role of the medial amygdaloid nucleus Neuroscience 1997 78 1027 35 10.1016/S0306-4522(96)00629-X 9174071

[B23] Wood RI Newman SW Integration of chemosensory and hormonal cues is essential for mating in the male Syrian hamster J Neurosci 1995 15 7261 9 7472480 10.1523/JNEUROSCI.15-11-07261.1995 PMC6578098

[B24] Lim MM Hammock EA Young LJ The role of vasopressin in the genetic and neural regulation of monogamy J Neuroendocrinol 2004 16 325 32 10.1111/j.0953-8194.2004.01162.x 15089970

[B25] Bluthe RM Schoenen J Dantzer R Androgen-dependent vasopressinergic neurons are involved in social recognition in rats Brain Res 1990 519 150 7 10.1016/0006-8993(90)90073-K 1975762

[B26] Ho JM Murray JH Demas GE Goodson JL Vasopressin cell groups exhibit strongly divergent responses to copulation and male-male interactions in mice Horm Behav 2010 58 368 77 10.1016/j.yhbeh.2010.03.021 20382147 PMC4195792

[B27] Choi GB Dong H-w Murphy AJ Valenzuela DM Yancopoulos GD Swanson LW Anderson DJ Lhx6 delineates a pathway mediating innate reproductive behaviors from the amygdala to the hypothalamus Neuron 2005 46 647 60 10.1016/j.neuron.2005.04.011 15944132

[B28] Samuelsen CL Meredith M The vomeronasal organ is required for the male mouse medial amygdala response to chemical-communication signals, as assessed by immediate early gene expression Neuroscience 2009 164 1468 76 10.1016/j.neuroscience.2009.09.030 19778594 PMC2801006

[B29] Meredith M Vomeronasal, olfactory, hormonal convergence in the brain. cooperation or coincidence? Ann N Y Acad Sci 1998 855 349 61 10.1111/j.1749-6632.1998.tb10593.x 9929627

[B30] Morris JA Jordan CL King ZA Northcutt KV Breedlove SM Sexual dimorphism and steroid responsiveness of the posterodorsal medial amygdala in adult mice Brain Res 2008 1190 115 21 18054901 10.1016/j.brainres.2007.11.005 PMC2258085

[B31] Blake CB Meredith M Change in number and activation of androgen receptor-immunoreactive cells in the medial amygdala in response to chemosensory input Neuroscience 2011 190 228 38 21684322 10.1016/j.neuroscience.2011.05.056 PMC3156313

[B32] Zhou L Blaustein JD De Vries GJ Distribution of androgen receptor immunoreactivity in vasopressin- and oxytocin-immunoreactive neurons in the male rat brain Endocrinology 1994 134 2622 7 8194487 10.1210/endo.134.6.8194487

[B33] Kondo Y Arai Y Functional association between the medial amygdala and the medial preoptic area in regulation of mating behavior in the male rat Physiol Behav 1995 57 69 73 10.1016/0031-9384(94)00205-J 7878127

[B34] Been LE Petrulis A Chemosensory and hormone information are relayed directly between the medial amygdala, posterior bed nucleus of the stria terminalis, and medial preoptic area in male Syrian hamsters Horm Behav 2011 59 536 48 10.1016/j.yhbeh.2011.02.005 21316366 PMC3081384

[B35] Johnston JB Further contributions to the study of the evolution of the forebrain J Comp Neurol 1923 35 337 481 10.1002/cne.900350502

[B36] Kabelik D Morrison JA Goodson JL Cryptic regulation of vasotocin neuronal activity but not anatomy by sex steroids and social stimuli in opportunistic desert finches Brain Behav Evol 2010 75 71 84 10.1159/000297522 20332615 PMC2914398

[B37] Baum MJ Tobet SA Starr MS Bradshaw WG Implantation of dihydrotestosterone propionate into the lateral septum or medial amygdala facilitates copulation in castrated male rats given estradiol systemically Horm Behav 1982 16 208 23 10.1016/0018-506X(82)90020-4 7118087

[B38] Dluzen DE Muraoka S Engelmann M Landgraf R The effects of infusion of arginine vasopressin, oxytocin, or their antagonists into the olfactory bulb upon social recognition responses in male rats Peptides 1998 19 999 1005 10.1016/S0196-9781(98)00047-3 9700747

[B39] Tobin VA Hashimoto H Wacker DW Takayanagi Y Langnaese K Caquineau C Noack J Landgraf R Onaka T Leng G Meddle SL Engelmann M Ludwig M An intrinsic vasopressin system in the olfactory bulb is involved in social recognition Nature 2010 464 413 7 10.1038/nature08826 20182426 PMC2842245

[B40] Wacker DW Ludwig M Vasopressin, oxytocin, and social odor recognition Horm Behav 2012 61 259 65 10.1016/j.yhbeh.2011.08.014 21920364

[B41] Goodson JL Kelly AM Kingsbury MA Evolving nonapeptide mechanisms of gregariousness and social diversity in birds Horm Behav 2012 61 239 50 10.1016/j.yhbeh.2012.01.005 22269661 PMC3312996

[B42] Garrison JL Macosko EZ Bernstein S Pokala N Albrecht DR Bargmann CI Oxytocin/vasopressin-related peptides have an ancient role in reproductive behavior Science 2012 338 540 3 10.1126/science.1226201 23112335 PMC3597094

[B43] Young LJ Nilsen R Waymire KG MacGregor GR Insel TR Increased affiliative response to vasopressin in mice expressing the V1a receptor from a monogamous vole Nature 1999 400 766 8 10.1038/23475 10466725

[B44] Raab A Haedenkamp G Impact of social conflict between mice on testosterone binding in the central nervous system Neuroendocrinology 1981 32 272 7 10.1159/000123172 6113558

[B45] Blanchard DC Sakai RR McEwen B Weiss SM Blanchard RJ Subordination stress: behavioral, brain, and neuroendocrine correlates Behav Brain Res 1993 58 113 21 10.1016/0166-4328(93)90096-9 8136039

[B46] Cooke BM Chowanadisai W Breedlove SM Post-weaning social isolation of male rats reduces the volume of the medial amygdala and leads to deficits in adult sexual behavior Behav Brain Res 2000 117 107 13 10.1016/S0166-4328(00)00301-6 11099763

[B47] Van Zwieten EJ Kos WT Ravid R Swaab DF Decreased number of vasopressin immunoreactive neurons in the medial amygdala and locus coeruleus of the aged rat Neurobiol Aging 1993 14 245 8 10.1016/0197-4580(93)90008-Y 8321392

[B48] Lin D Boyle MP Dollar P Lee H Lein ES Perona P Anderson DJ Functional identification of an aggression locus in the mouse hypothalamus Nature 2011 470 221 6 10.1038/nature09736 21307935 PMC3075820

